# An exploratory study on cortical hemodynamics and handgrip strength phenotypes in long-term hospitalized patients with stable schizophrenia

**DOI:** 10.1371/journal.pone.0349442

**Published:** 2026-05-15

**Authors:** Yanping Feng, Yilin Wang, Binyou Wang

**Affiliations:** Department of Psychiatry, Zigong Mental Health Center, the Zigong Affiliated Hospital, Southwest Medical University‌‌, Zigong, Sichuan, China; University of Thessaly Faculty of Medicine: Panepistemio Thessalias Tmema Iatrikes, GREECE

## Abstract

**Objective:**

This exploratory study aims to investigate the relationship between handgrip strength (HGS) phenotypes and cortical hemodynamics in long-term hospitalized patients with stable schizophrenia, and to evaluate whether HGS could serve as a potential indicator of neurocognitive function in this population.

**Methods:**

In this cross-sectional study, 96 patients with stable schizophrenia were enrolled. HGS was assessed using a calibrated dynamometer. Cortical hemodynamic responses during a Verbal Fluency Task (VFT) were measured using functional near-infrared spectroscopy (fNIRS), with metrics derived from both oxygenated hemoglobin (Oxy-Hb) and total hemoglobin (Total-Hb) signals, including the S-integral, T-centroid, and K-activation. Statistical analyses comprised Spearman correlation analysis, univariate linear regression, and general linear models with post hoc comparisons.

**Results:**

Among the participants, 40.60% (39/96) were male and 59.40% (57/96) were female, with a median (P25, P75) age of 52 (42, 56) years. Univariate linear regression revealed a significant positive association between HGS asymmetry and frontal lobe S-integral values (Oxy-Hb: β = 65.650, 95% CI: 18.488–112.812, P = 0.007; Total-Hb: β = 64.878, 95% CI: 19.360–110.395, P = 0.006). However, after adjusting for potential confounders, general linear models revealed no significant differences in frontal S-integral values across HGS asymmetry subgroups, regardless of whether asymmetry was classified by percentage-based thresholds or dominance type (all Bonferroni-adjusted P > 0.05).

**Conclusion:**

In this exploratory study, HGS asymmetry was associated with frontal S-integral values unadjusted; however, after adjusting for confounders and multiple comparisons, no significant independent association remained. HGS phenotypes cannot be considered reliable or simple surrogate markers of neurocognitive activity in this population, underscoring the substantial influence of confounding neurophysiological factors in schizophrenia.

## Introduction

Schizophrenia is a heterogeneous psychiatric disorder defined by three core symptom domains: positive symptoms, such as hallucinations and delusions; negative symptoms, including social withdrawal and alogia; and cognitive impairments, including deficits in processing speed and executive function [1–3]. Due to its chronic and relapsing course, schizophrenia often requires long-term clinical management and, in many cases, prolonged inpatient hospitalization, placing a considerable burden on affected individuals, their families, and healthcare systems [4,5]. Although psychopharmacological treatments have advanced, the implementation of individualized therapeutic strategies remains limited by the complex, multifactorial pathophysiology of the disorder and the lack of validated biomarkers for clinical stratification and prognosis.

Handgrip strength (HGS) has gained attention as an objective and reproducible indicator of neuromuscular function [6]. Beyond its traditional use as a marker of physical health, HGS is increasingly recognized as a predictor of cognitive decline and dementia in older adults [7–9], as well as a surrogate measure linked to brain structure and function. The relationship between HGS and cognition is thought to reflect shared neural substrates, given that both motor performance and cognitive processes depend on the coordinated functioning of central nervous system networks [10,11]. Consistent with this notion, neuroimaging studies have demonstrated associations between HGS and gray matter volume in both cortical and subcortical regions [12,13]. Moreover, atypical HGS phenotypes, particularly handgrip asymmetry between the dominant and non-dominant hands, may indicate hemispheric imbalance or disrupted interhemispheric communication [14] and have been independently associated with adverse cognitive outcomes and increased risk of neurodegeneration in the general population [15].

Individuals with long-term hospitalization for schizophrenia are exposed to compounded risks of motor and cognitive impairment arising from the underlying disease process, prolonged antipsychotic treatment, and institutional factors such as physical inactivity and environmental deprivation [16–18]. Previous studies in schizophrenia have reported reduced HGS and associations between lower grip strength and poorer cognitive performance [19,20]. Further, a recent study suggests a potential association between HGS phenotypes and cortical hemodynamic activity [21]. However, it remains unclear whether specific HGS characteristics, including absolute strength and inter-hand asymmetry, are related to task-evoked neural activity underlying discrete cognitive domains, such as language production and executive function, in this clinically vulnerable population.

Therefore, this exploratory study aimed to examine the association between HGS phenotypes and functional neurophysiological alterations in the frontal and temporal cortices of long-term hospitalized patients with stable schizophrenia. Using functional near-infrared spectroscopy (fNIRS) during a verbal fluency task (VFT), we assessed the relationships between HGS, HGS asymmetry, and task-related cortical hemodynamic responses. Elucidating these associations may clarify whether HGS represents a feasible peripheral marker of neurophysiological status in schizophrenia and enhance understanding of the neural mechanisms linking physical and cognitive dysfunction in this disorder.

## Methods

### Study design and participants

This cross-sectional study was conducted at the Zigong Mental Health Center from August 1 to 15 September 2024, in accordance with the Declaration of Helsinki.

Patients were eligible if they: 1) age 18 years or older; (2) a confirmed diagnosis of schizophrenia based on ICD-10 diagnostic criteria; 3) a clinically stable condition, defined by sustained symptom control, absence of symptom exacerbation for more than one month, and a stable antipsychotic treatment regimen for at least two months before enrollment; 4) a duration of hospitalization of at least three months; 5) willingness to participate with provision of written informed consent; and (6) capacity to complete all study assessments. Exclusion criteria included: (1) the presence of autoimmune diseases or receipt of ongoing anti-cancer therapy; (2) severe hepatic or renal impairment; and (3) inability to undergo handgrip strength assessment or functional near-infrared spectroscopy testing, or to complete the required psychopathological and cognitive evaluations.

### Informed consent process‌‌

This investigation strictly followed the Declaration of Helsinki, and received ethical approval from received ethical approval from the Zigong Mental Health Center in China (IRB2023024). In light of the participants’ potential cognitive vulnerability, a structured two-stage consent procedure was implemented. First, surrogate consent was secured from the legal guardian of each participant. Following a comprehensive telephone explanation of the study’s purpose, procedures, risks, benefits, and data handling—with emphasis that no invasive or chargeable procedures beyond standard care were involved—written informed consent was obtained from the guardian. Subsequently, the attending physician conducted an in-person meeting with the patient. The information was conveyed in simplified, non-technical language, and the patient’s understanding was assessed clinically through interactive dialogue and direct questioning rather than via a formal tool (e.g., MacCAT-CR). Key concepts such as voluntary participation and the right to withdraw were explicitly reviewed and confirmed. Only after verifying adequate comprehension and voluntary willingness did the patient provide written consent (signature or fingerprint).

All original signed consent documents were archived with the IRB and filed with the hospital’s Medical Administration Department. This dual-consent framework was maintained during the 2024 follow-up, which introduced the fNIRS assessment. For continuing participants, the original consent was reviewed, and the new procedure was re-explained verbally, with ongoing understanding and willingness confirmed prior to testing.

Regarding data collection procedures, the timing of assessments was carefully synchronized with participants’ routine clinical care and physical examination schedules to reduce participant burden. Specifically, among the 122 patients enrolled in 2023 who remained in follow-up (excluding those discharged, transferred, or withdrawn), researchers coordinated the data collection timeline around participants’ scheduled fNIRS examinations. All anthropometric measurements and scale assessments were conducted one to two days prior to the fNIRS testing. Furthermore, to ensure robust data integrity, any missing demographic or clinical details identified within the electronic medical records were promptly verified and supplemented through direct communication with participants on the day of their fNIRS testing. Among the 122 patients with stable schizophrenia initially recruited, 26 were excluded because of incomplete medication dosage information (n = 23) or missing PANSS data (n = 3). Therefore, 96 participants were included in the final analysis.

### HGS assessment

HGS was assessed using a calibrated hydraulic dynamometer (EH101; Camry, Guangdong, China; measurement range: 0–90.0 kg). Hand dominance was determined by participant self-report. We measured HGS using the standing posture protocol from National Health and Nutrition Examination Survey (NHANES). According to the NHANES protocol [22], participants squeezed the dynamometer maximally with the second metacarpophalangeal joint at approximately 90°, elbows fully extended, shoulders adducted within ~10°, head upright, wrists neutral, and feet parallel at hip-width. However, given that our study population consisted of long-term hospitalized patients with schizophrenia who presented with cognitive impairment, many of whom had obesity due to antipsychotic medication use and physical inactivity, researchers provided manual assistance to stabilize their standing posture prior to each trial, aiming to adhere as closely as possible to the NHANES protocol.

Three trials were completed for each hand, beginning with the non-dominant hand, with a 2-minute rest interval between trials to minimize fatigue. The maximum value (kg) obtained across all trials for each hand was recorded for analysis. All measurements were performed under controlled environmental conditions.

### fNIRS indicators measurement

fNIRS enables non-invasive monitoring of cortical hemodynamics by detecting changes in near-infrared light absorption by oxygenated hemoglobin (Oxy-Hb) and deoxygenated hemoglobin (Deoxy-Hb) in superficial cortical layers [23]. Demonstrating good concordance with functional magnetic resonance imaging (fMRI) [24] and offering practical advantages such as portability and relative tolerance to movement, fNIRS is particularly well-suited for research in populations with limited mobility, including long-term hospitalized patients [25]. The technique shows robust test–retest reliability across sessions and channels, high reproducibility of group-level measurements in superficial cortical regions [26], and high temporal resolution, allowing real-time assessment of dynamic oxygenation changes during both task-based and resting conditions [27]. Furthermore, via specific hardware designs and advanced signal processing, fNIRS effectively distinguishes cortical hemodynamic responses from extracerebral artifacts (e.g., cardiac pulsation, scalp blood flow) [28]. These features make fNIRS a flexible tool for capturing rapid neural dynamics.

**Fig 1:**
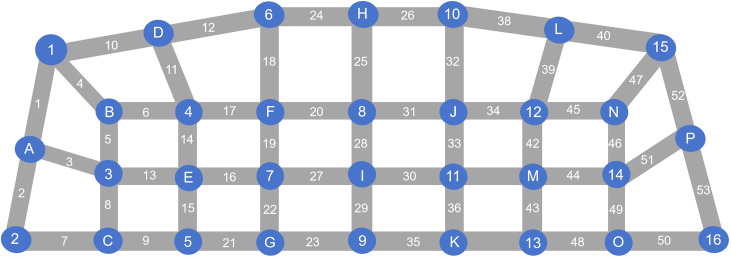
Optode arrangement for fNIRS sensor over the frontal and temporal region.

Cortical hemodynamic data were acquired using the BS-3000L fNIRS system (Wuhan Znion Technology Co., China) in a quiet, controlled environment. The system consisted of 16 light emitters and 16 detectors operating at 690 and 830 nm, respectively, with a sampling rate of 20 Hz. Optodes were arranged with an inter-optode distance of 3 cm to cover bilateral frontal and temporal regions, yielding a total of 53 measurement channels (see Fig 1 for optode configuration). The lowest probe was positioned along the T4–Fpz–T3 line according to the International 10–20 system [29] and secured using a head strap.

**Fig 2:**
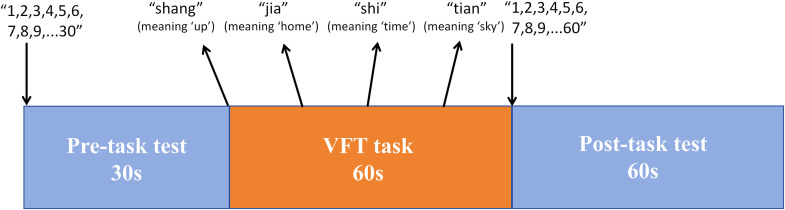
The process of VFT.

Participants completed a standardized Verbal Fluency Task (VFT) (Fig 2), which comprised a 30-s pre-task baseline (counting from 1 to 30), a 60-s task phase during which participants generated as many Chinese phrases as possible using visually presented characters that changed every 15 s, and a 60-s post-task baseline (counting from 1 to 60).

### Signal processing and indicator extraction

Signal preprocessing was performed using the Homer2 toolbox (implemented in MATLAB; MathWorks, Natick, MA, USA) [30] integrated into the fNIRS system. The pipeline included the following steps. First, raw data quality was screened by calculating the coefficient of variation (CV) across the entire recording for each channel; a CV threshold of 35% was applied, and participants with >80% of channels meeting this criterion were retained for further analysis. Retained data were converted to optical density (OD). Motion artifacts were then corrected using a spline interpolation method [31], where signal changes exceeding 20% of the standard deviation within 3 s were identified as artifacts. Next, a 0.01 Hz high‑pass filter was applied to remove baseline drift, and a 0.1 Hz low‑pass filter was used to attenuate high‑frequency physiological noise from cardiac pulsation, respiration, blood pressure fluctuations, and superficial blood flow [32]. The filtered signals were then converted into concentration changes of Oxy-Hb, Deoxy-Hb, and total hemoglobin (Total-Hb = Oxy-Hb + Deoxy-Hb) using the modified Beer–Lambert law [33]. Task-related activation was calculated as the mean hemoglobin concentration change during the task period relative to a 10-s pre-task baseline. Cortical registration of fNIRS channels was performed according to the probe arrangement based on the International 10–20 system. Channels overlying bilateral temporal regions (ch2, ch3, ch5, ch7, ch8, ch13, ch44, ch46, ch49, ch50, ch51, and ch53) were designated as temporal lobe measurements, whereas the remaining channels were classified as frontal lobe responses.

For the fNIRS data analysis, we focused on Oxy-Hb signals, as they reflect cortical activation more directly than Deoxy-Hb [34–36], and analyzed Total-Hb to assess regional cerebral blood volume changes [37]. Three principal hemodynamic metrics were extracted from the Oxy-Hb and Total-Hb response curves for both frontal and temporal regions: (1) S-integral, defined as the area under the response curve during the task period and representing the overall magnitude of neural activation [38], (2) T-centroid, corresponding to the time point at which 50% of the cumulative positive hemodynamic response is reached, reflecting the temporal distribution of activation [39]; and (3) K-activation, calculated as the linear slope of the initial ascending phase (first 5 s) of the response curve, indicating the speed of cortical activation following task onset [40].

### Psychopathological and cognitive function evaluation

Assessments were conducted by trained psychiatrists using standardized clinical instruments:

Psychiatric symptoms: Psychopathology was evaluated with the 30-item Positive and Negative Syndrome Scale (PANSS) [41,42]. Each item is scored on a 7-point Likert scale ranging from 1 (absent) to 7 (extreme), with higher total scores reflecting greater symptom severity. As referenced in previous work [43], total scores of 58, 75, and 95 correspond to mild, moderate, and marked levels of illness severity, respectively.

Depressive and anxiety symptoms: Depressive symptoms and anxiety were assessed using the 9-item Patient Health Questionnaire (PHQ-9) [44] and the 7-item Generalized Anxiety Disorder scale (GAD-7) [45], respectively. Scores of 5 or higher on either scale were considered indicative of clinically relevant symptoms [46].

Global cognitive function: Overall cognitive performance was measured with the Chinese version of the Montreal Cognitive Assessment (MoCA-C) [47], a 30-point screening tool in which lower scores denote greater cognitive impairment. The MoCA-C has demonstrated good validity and applicability in patients with schizophrenia [48], with scores of 25–27 used to identify mild cognitive deficits, and scores of 24 or below indicating severe cognitive deficits [48].

### Covariates

Potential confounding variables were collected through structured interviews and review of electronic medical records. These included demographic factors such as age, sex, body mass index (BMI), marital status, educational attainment, self-reported sleep quality, visual or hearing impairment, and tobacco and alcohol use. Clinical characteristics were also recorded, including history of psychiatric symptom relapse, family history of mental disorders, first-episode status, duration of illness (years), hospitalized time (months), falls history, chlorpromazine-equivalent dosage (mg/day), history of hospital bacterial pneumonia, COVID-19 history, treatment method (antipsychotic monotherapy), and number of comorbid chronic diseases. Depressive status, anxiety status, and scores from the PANSS, GAD-7, and MoCA-C assessments were included as potential confounders.

### Statistical analyses

Data analysis was performed using SPSS software (version 25.0), with a two-sided P-value of < 0.05 considered to be statistically significant. Categorical variables are presented as frequencies and percentages. The distribution of continuous variables was evaluated using the Shapiro–Wilk test. Normally distributed data are expressed as mean ± standard deviation (SD), whereas non-normally distributed data are reported as medians with interquartile ranges (IQRs), defined by the 25th (P25) and 75th (P75) percentiles.

Spearman’s Rho correlation was employed to assess associations between HGS phenotypes, including absolute HGS and HGS asymmetry, and fNIRS-derived hemodynamic indicators (S-integral, T-centroid, and K-activation). Following the identification of a significant correlation between HGS asymmetry and frontal S-integral, univariate linear regression analyses were performed to further examine this relationship.

To compare frontal S-integral values across different HGS asymmetry categories, participants were grouped using two distinct classification approaches derived from established literature. Method A (percentage-based classification) [49,50] classifies asymmetry as ≤10%, 10.1–20%, or >20%. Method B (dominance-based classification) [51,52] defines non-asymmetry as a non-dominant-to-dominant HGS ratio of 0.9–1.1, dominant asymmetry as a ratio <0.9, and non-dominant asymmetry as a ratio >1.1. Univariate general linear models were employed separately for each classification scheme to examine the association between HGS asymmetry subgroups and frontal S-integral values, with all covariates entered simultaneously into the model. Based on prior evidence indicating potential influences on cerebral hemodynamics [35,53,54], the following covariates were forced into the regression analyses: age, sex, chlorpromazine-equivalent dose, disease duration, BMI, MoCA-C scores, PANSS scores, education level, smoking history, and drinking history. Multicollinearity among the independent variables in these models was assessed using the variance inflation factor (VIF); a VIF value below 5 signified no serious collinearity [55]. To control for multiple testing, a Bonferroni correction was applied, and adjusted p-values are reported accordingly. This conservative approach was adopted because of the limited number of pairwise comparisons involved and our aim to minimize Type I error, especially given the modest sample size and the preliminary nature of the study.

## Results

### Participant characteristics

The demographic, clinical, and hemodynamic profiles of the cohort are detailed in Table 1. Of the 96 patients, 40.60% (39/96) were male and 59.40% (57/96) were female, with a median (P25, P75) age of 52 (42, 56) years. The medians of absolute HGS and the asymmetry ratio were 20.55 (14.65, 28.25) kg and 0.94 (0.85, 1.07), respectively.For fNIRS indicators, median (P25, P75) values were as follows: in the frontal lobe, S-integral values were 15.22 (5.35, 43.35) for Oxy‑Hb and 12.55 (4.8, 31.0) for Total‑Hb; K‑activation values were 0.0003 (−0.0003, 0.0009) for Oxy‑Hb and 0.0002 (−0.0003, 0.0009) for Total‑Hb. In the temporal lobe, S‑integral values were 22.27 (−1.73, 48.28) for Oxy‑Hb and 17.2 (−13.5, 38.8) for Total‑Hb; K‑activation values were 0.0004 (−0.0003, 0.0009) for Oxy‑Hb and 0.0005 (−0.0001, 0.001) for Total‑Hb. Mean ± SD values for T‑centroid were as follows: frontal lobe, 54.54 ± 16.24 (Oxy‑Hb) and 53.83 ± 16.18 (Total‑Hb); temporal lobe, 54.66 ± 16.18 (Oxy‑Hb) and 51.29 ± 14.92 (Total‑Hb).

**Table 1:** Demographic and clinical characteristics of patients with stable schizophrenia.

Characteristics		Total participants (N = 96)
**Demographic**		
Age(years), median(P25,P75)		52(42,56)
Male sex, n(%)		39(40.6)
BMI(kg/m2), mean±SD		25.42 ± 3.88
Marital status (married), n(%)		24(25)
Education,n(%)	Illiterate	7(7.3)
	High school and below	84(87.5)
	University and above	5(5.2)
Vision impairment (Yes), n(%)		4(4.2)
Hearing impairment (Yes), n(%)		0(0)
Substance use history (Yes), n(%)	Smoking	28(29.2)
	Alcohol use	7(7.3)
Sleep quality during last month(Poor/Very Bad), n(%)		78(81.3)
**Clinical**		
Family history of mental disorder (Yes), n(%)		30(31.3)
First episode (Yes), n(%)		2(2.1)
Disease duration (years),median(P25,P75)		21.50(12.25,31.0)
Hospitalized time(months),median(P25,P75)		56.5(28.25,114.25)
Falls history (Yes), n(%)		14(14.6)
History of hospital bacterial pneumonia(Yes), n(%)		9(9.4)
COVID-19 infection history(Yes), n(%)		8(8.3)
Relapse of Psychiatric Symptoms history(Yes), n(%)		8(8.3)
Treatment method (antipsychotic monotherapy), n(%)		87(90.6)
Chlorpromazine equivalent dose (mg/day), mean±SD		499.32 ± 218.37
Chronic diseases(≥1), n(%)		71(74)
MoCA-C scores,median (P25,P75)		19.0(13.0,25.0)
Mild cognitive deficits, n(%)		11(11.5)
Severe cognitive deficits, n(%)		68(70.8)
PANSS scores，mean ± SD		55 ± 11
Mildly ill, n(%)		58(60.4)
Moderately ill, n(%)		34(35.4)
Markedly ill, n(%)		4(4.2)
PHQ-9 Scores, median(P25,P75)		0(0,2)
Depressive Symptoms, n(%)		5(5.2)
GAD-7 Scores, median(P25,P75)		0(0,0)
Anxiety, n(%)		0(0)
Handgrip strength value (kg), median(P25,P75)		20.55(14.65,28.25)
Handgrip strength asymmetry value, median(P25,P75)		0.94(0.85,1.07)
**fNIRS metrics**		
Oxy-Hb(Frontal Lobe)	S-integral value, median(P25,P75)	15.22(5.35,43.35)
	T-centroid value, mean±SD	54.54 ± 16.24
	K-activation value, median(P25,P75)	0.0003(−0.0003,0.0009)
Total-Hb(Frontal Lobe)	S-integral value, median(P25,P75)	12.55(4.83,31.04)
	T-centroid value, mean±SD	53.83 ± 16.18
	K-activation value, median(P25,P75)	0.0002(−0.0003,0.0009)
Oxy-Hb(Temporal Lobe)	S-integral value, median(P25,P75)	22.27(−1.73,48.28)
	T-centroid value, mean±SD	54.66 ± 16.18
	K-activation value, median(P25,P75)	0.0004(−0.0003,0.0009)
Total-Hb(Temporal Lobe)	S-integral value, median(P25,P75)	17.18(−13.50,38.79)
	T-centroid value, mean±SD	51.29 ± 14.92
	K-activation value, median(P25,P75)	0.0005(−0.0001,0.001)

PHQ-9, Patient Health Questionnaire-9; GAD-7, Generalized Anxiety Disorder-7; MoCA-C, Montreal Cognitive Assessment-Chinese version; PANSS, Positive and Negative Syndrome Scale.

Mild cognitive deficits, 25 ≤ MoCA-C scores ≤ 27; Severe cognitive deficits, MoCA-C scores ≤ 24; Mildly ill, PANSS scores ≤ 57; Moderately ill, 58 ≤ PANSS scores ≤ 74; Markedly ill, 75 ≤ PANSS scores ≤ 94; Depressive Symptoms, PHQ-9 Scores ≥ 5; Anxiety, GAD-7 Scores ≥ 5.

### Correlations between HGS phenotypes and fNIRS indicators

Spearman’s correlation analysis revealed a significant positive association between HGS asymmetry and the frontal lobe S-integral value, observed for both Oxy-Hb (r = 0.281, P < 0.01) and Total-Hb (r = 0.251, P < 0.05) signals (Table 2). No other significant relationships were identified between HGS phenotypes (absolute strength or asymmetry) and any additional fNIRS-derived indices, including T-centroid or K-activation, in either the frontal or temporal lobes (all P > 0.05).

**Table 2:** Correlation between HGS value, HGS asymmetry value and fNIRS indicators in the frontal and temporal lobes.

Variable	Oxy-Hb						Total-Hb					
	Frontal Lobe			Temporal Lobe			Frontal Lobe			Temporal Lobe		
	S-integral value	T-centroid value	K-activation value	S-integral value	T-centroid value	K-activation value	S-integral value	T-centroid value	K-activation value	S-integral value	T-centroid value	K-activation value
Handgrip strength	−0.016	0.026	0.183	0.115	0.107	0.175	−0.02	0.02	0.175	0.032	−0.043	0.135
Handgrip strength asymmetry	0.281^**^	0.159	−0.065	0.031	0.006	−0.029	0.251^*^	0.097	−0.043	0.075	0.122	−0.004

*, P < 0.05; **, P < 0.01.

### Correlations between HGS asymmetry and S-integral value of the frontal lobe

Univariate linear regression analysis was conducted with the frontal S-integral as the dependent variable. HGS asymmetry was significantly and positively associated with frontal S-integral values for both Oxy-Hb (β = 65.650, 95% CI: 18.488–112.812, P = 0.007) and Total-Hb (β = 64.878, 95% CI: 19.360–110.395, P = 0.006) signals (Table 3). In comparison, none of the assessed demographic or clinical variables showed significant associations with the frontal S-integral (all P > 0.05). In contrast, as T-centroid and K-activation showed no significant associations in unadjusted analyses (Table 2), they were not further analyzed in multivariable models.

**Table 3:** Univariate analysis of HGS asymmetry, general information, clinical characteristics and S-integral value of frontal lobe among inpatients with stable schizophrenia.

Variable	HbO_2_-S-integral value		HbT-S-integral value	
	β(95%CI)	P-value	β(95%CI)	P-value
Handgrip strength asymmetry	65.650(18.488,112.812)	**0.007**	64.878(19.360,110.395)	**0.006**
**Demographic**				
Age	0.007(−0.713,0.727)	0.984	−0.101(−0.797,0.595)	0.774
Sex ^a^	−5.890(−21.776,9.995)	0.463	−9.098(−24.389,6.193)	0.24
BMI	−0.648(−2.670,1.374)	0.526	−0.719(−2.673,1.235)	0.467
Marital status ^b^	3.901(−14.151,21.953)	0.669	10.668(−6.666,28.002)	0.225
Education ^c^	14.828(−7.132,36.788)	0.183	13.461(−7.796,34.718)	0.212
Vision impairment ^d^	3.071(−36.080,42.221)	0.877	1.585(−36.273,39.443)	0.934
Smoking ^e^	−1.159(−18.372,16.053)	0.894	2.772(−13.862,19.407)	0.741
Alcohol use ^f^	−2.712(−32.800,27.377)	0.858	3.791(−25.296,32.878)	0.796
Sleep quality during last month ^g^	−10.378(−26.400,5.643)	0.202	−6.878(−22.441,8.686)	0.382
**Clinical**				
Family psychiatric history ^h^	−2.233(−19.107,14.642)	0.793	4.808(−11.484,21.100)	0.559
First episode ^i^	4.291(−50.484,59.066)	0.877	−0.359(−53.328,52.610)	0.989
Disease duration	−0.013(−0.727,0.702)	0.972	0.119(−0.571,0.810)	0.732
Hospitalized time	0.052(−0.062,0.165)	0.369	0.037(−0.073,0.147)	0.506
History of hospital bacterial pneumonia ^j^	2.439(−24.400,29.277)	0.857	−5.074(−31.008,20.860)	0.699
COVID-19 infection history ^k^	−4.762(−33.055,23.530)	0.739	−7.490(−34.819,19.840)	0.588
Relapse of Psychiatric Symptoms history ^l^	20.818(−7.169,48.805)	0.143	13.617(−13.612,40.847)	0.323
Treatment method ^m^	−5.804(−44.226,34.058)	0.797	−6.244(−44.082,31.594)	0.744
Chlorpromazine equivalent dose	−0.002(−0.038,0.034)	0.912	0.000(−0.035,0.035)	0.987
Chronic diseases ^n^	6.888(−11.986,25.763)	0.470	9.719(−8.472,27.911)	0.291
Falls history ^o^	10.302(−11.767,32.370)	0.356	−0.596(−22.031,20.839)	0.956
MoCA-C	0.934(−0.120,1.989)	0.082	0.771(−0.253,1.795)	0.138
PANSS scores	−0.047(−0.795,0.701)	0.901	0.244(−0.478,0.966)	0.504
Depressive Symptoms ^p^	−22.027(−56.950,12.897)	0.214	−19.982(−53.783,13.819)	0.243

HGS, handgrip strength, MoCA-C, Montreal Cognitive Assessment-Chinese version; PANSS, Positive and Negative Syndrome Scale.

^a^Male, ^b^ married, ^c^ illiterate, ^d^ no vision problems, ^e^ no smoke history, ^f^ no drink history, ^g^ good sleep quality during last month, ^h^ no family psychiatric history, ^i^ non-first episode, ^j^ no hospital bacterial pneumonia infection history, ^k^ no COVID-19 infection history, ^l^ no relapse of psychiatric symptoms history, ^m^ antipsychotic monotherapy, ^n^ no chronic diseases, ^o^ no history of falls, ^p^ without depressive symptoms were used as reference.

### Multiple comparisons of S-integral value across HGS asymmetry groups

When participants were classified according to percentage-based asymmetry thresholds (Method A), univariate general linear models adjusting for predefined covariates (age, sex, chlorpromazine-equivalent dose, disease duration, BMI, MoCA-C scores, PANSS scores, education level, smoking history, and drinking history) revealed no significant differences in frontal S-integral values among the ≤ 10%, 10.1–20%, and >20% asymmetry groups for either Oxy-Hb or Total-Hb signals (all adjusted P > 0.05; Table 4). Similarly, classification based on dominance type (Method B) showed no statistically significant differences in frontal S-integral values across the non-asymmetry, dominant asymmetry, and non-dominant asymmetry groups after Bonferroni correction (all adjusted P > 0.05). Multicollinearity diagnostics confirmed no serious collinearity among independent variables in either model, with all VIF values below 5 (Supplementary Table 1).

**Table 4:** Multiple comparisons of the S-integral value of the frontal lobe by different grouping methods of HGS asymmetry.

Part	N(%)	Group A	Group B	Oxy-Hb					Total-Hb				
				S-integral value,median (P25,P75)	Mean difference (A – B)	SE	P-value	95% CI	S-integral value,median (P25,P75)	Mean difference (A – B)	SE	P-value	95% CI
A	≤10% asymmetry, 38(39.58)	≤10% asymmetry	10–20% asymmetry	18.40(5.76,47.36)	10.123	9.118	0.81	−12.157,32.402	13.95(4.84,44.10)	13.982	8.688	0.334	−7.246,35.210
	10.1–20% asymmetry, 40(41.67)	≤10% asymmetry	>20% asymmetry	15.16(5.05,37.80)	−1.041	11.622	1	−29.437,27.357	11.86(2.17,25.14)	−2.970	11.073	1	−30.027,24.087
	>20% asymmetry, 18(18.75)	10–20% asymmetry	>20% asymmetry	15.17(4.67,56.4)	−11.162	11.443	0.996	−39.123,16.798	13.26(6.97,38.83)	−16.952	10.903	0.371	−43.593,9.690
B	Non-asymmetry, 38(39.58)	Non-asymmetry	Dominant asymmetry	18.40(5.76,47.36)	14.639	8.988	0.321	−7.323,36.601	13.95(4.84,44.10)	16.955	8.603	0.156	−4.067,37.976
	Dominant asymmetry, 40(41.67)	Non-asymmetry	Non-dominant asymmetry	12.95(4.24,30.67)	−10.919	11.494	1	−39.005,17.166	11.13(2.17,22.43)	−9.262	11.002	1	−36.145,17.620
	Non-dominant asymmetry, 18(18.75)	Dominant asymmetry	Non-dominant asymmetry	29.36(12.34,64.78)	−25.558	11.576	0.09	−53.844,2.727	20.76(7.32,54.04)	−26.127	11.080	0.061	−53.291,0.857

Part A: grouping by asymmetry percentage thresholds; Part B: grouping by dominance type: non-dominant/dominant handgrip strength = 0.9–1.1 (non-asymmetry); non-dominant/dominant handgrip strength <0.9 (dominant asymmetry); non-dominant/dominant handgrip strength > 1.1 (non-dominant asymmetry). SE, standard error; CI, confidence interval. *Significant at P < 0.05. P-values shown in the table are Bonferroni-corrected p-values for multiple comparisons.

## Discussion

This exploratory investigation is, to our knowledge, the first to examine the relationship between HGS phenotypes and task-evoked cortical hemodynamic responses in long-term hospitalized patients with stable schizophrenia. The principal finding is that in unadjusted analyses, HGS asymmetry, rather than absolute HGS, showed a significant positive association with frontal lobe S-integral values during the VFT for both Oxy-Hb and Total-Hb signals. When HGS asymmetry was treated as a categorical variable and analyzed via two established classification approaches, no statistically significant differences in frontal S-integral values were observed across subgroups after covariate adjustment and Bonferroni correction for multiple comparisons. These results indicate that HGS phenotypes capture complex neuromuscular information and, in this clinical context, do not constitute a simple or direct surrogate of underlying neurocognitive activity.

The VFT is a well-established paradigm for probing frontotemporal function, and fNIRS offers a sensitive approach for detecting associated hemodynamic changes. Consistent with previous fNIRS studies in schizophrenia, which have repeatedly demonstrated frontal and temporal hypoactivation [38,56,57], our samples exhibited markedly attenuated hemodynamic responses. For example, the median frontal S-integral observed in this study (12.55 [4.8, 31.0]) was substantially lower than the mean value reported by Wei et al. (40.63 ± 56.52) in a broader, likely less chronic, schizophrenia sample [58]. This pronounced reduction in task-evoked activation may reflect the distinctive clinical characteristics of our population, including advanced age, prolonged illness duration, and the cumulative effects of long-term institutionalization and sustained antipsychotic exposure [59–63]. These factors are known to be associated with metabolic disturbances, reduced physical activity, and neuromuscular compromise, which may collectively exacerbate frontotemporal dysfunction through disrupted cortical–subcortical connectivity [64,65].

The observed positive association between HGS asymmetry and frontal S-integral values suggests a potential link between peripheral motor imbalance and central neurophysiological processes in schizophrenia. However, the absence of significant between-group differences in frontal S-integral values when HGS asymmetry was categorized by either percentage-based thresholds or dominance-based criteria highlights a critical methodological consideration. The commonly used cutoffs were developed and validated primarily in general or aging populations [49,50,52]. They may not be directly transferable to long-term hospitalized patients with schizophrenia, whose neuromuscular function is influenced by chronic illness, prolonged pharmacotherapy, and institutional factors. The modest sample size limited statistical power to detect between-group differences, and categorizing a continuous variable such as HGS asymmetry inevitably results in information loss, potentially obscuring associations evident in correlation-based analyses. These considerations emphasize the challenges of translating a peripheral motor measure into a categorical indicator of central neural function in this population.

Several limitations merit consideration. First, the relatively small sample size reduced statistical power for stratifying the cohort into clinically meaningful subgroups (e.g., based on age, illness duration, or comorbidity patterns) and for detecting group differences after correction for multiple comparisons. As a result, potentially important variation in how HGS relates to frontal lobe activation across different patient subtypes may have remained undetected. Second, the exclusive recruitment of long-term inpatients from a single institution resulted in a highly homogeneous sample, and the use of ICD-10 diagnostic criteria may further differ in phenotypic composition from studies applying DSM-5 classifications. Together, these factors substantially limit the generalizability of our findings to community-dwelling outpatients, acute-phase populations, or broader schizophrenia cohorts defined under different diagnostic frameworks. Third, informed consent capacity was assessed clinically without a standardized instrument (e.g., MacCAT‑CR), and consent was obtained by the treating physician, which may have introduced potential bias related to therapeutic misconception. Fourth, the use of manual assistance to stabilize standing posture during HGS measurement may limit comparability with standard data, and specific conditions that could directly affect HGS (e.g., upper-limb orthopedic disorders, primary myopathies, or peripheral neuropathies) were not systematically screened. Fifth, the MoCA‑C cutoff values used were derived from small samples of schizophrenia cohorts rather than validated, large‑scale normative data for this population, which may introduce potential misclassification of cognitive impairment in our study group. Finally, covariate control was limited, as medication effects were assessed primarily through chlorpromazine-equivalent dosing without detailed consideration of specific antipsychotic agents, precise dosages, or plasma drug levels. Other substances or concomitant medications known to affect neuromuscular performance or cerebral hemodynamics were also not comprehensively evaluated.

Future studies should utilize larger and more diverse cohorts and employ longitudinal designs to clarify the temporal direction of associations between motor and neurophysiological measures. Mechanistic investigations should incorporate multimodal neuroimaging, detailed molecular/neurochemical profiling, and multidimensional data such as comprehensive pharmacological analysis, structured physical health assessments (e.g., screening for upper limb-related orthopedic, myopathic, or neuropathic conditions), and cognitive evaluation. Additionally, future work should adopt independent consent assessors and validated capacity tools, and establish or validate population‑specific MoCA‑C cut‑offs for chronic schizophrenia. Validation of HGS phenotypes as practicable biomarkers further requires replication across clinically distinct schizophrenia populations, with intentional assessment of confounding factors including specific medication effects, comorbid somatic conditions, and activity profiles. This integrated approach will deepen the understanding of the mechanisms driving neuromuscular dysfunction in schizophrenia and ultimately inform the development of precise, stratified screening tools and personalized rehabilitation strategies.

## Conclusion

In conclusion, this exploratory study suggests that handgrip strength asymmetry demonstrates a complex, context-dependent relationship with frontal lobe activity in inpatients with stable schizophrenia, yet it cannot be readily reduced to categorical cutoffs or interpreted as a direct surrogate for neurocognitive function. These preliminary findings underscore the importance of integrated, multimodal biomarker approaches to more accurately characterize the underlying pathophysiology of schizophrenia and to inform the development of personalized clinical management strategies.

## Supporting information

S1 Table Variance inflation factor (VIF) for multicollinearity diagnostics of independent variables. (DOCX)

S1 File Data. (XLSX)
