# Supplementary material for: An exploratory study on cortical hemodynamics and handgrip strength phenotypes in long-term hospitalized patients with stable schizophrenia
Source: PLoS One. 2026 May 15;21(5):e0349442. doi: 10.1371/journal.pone.0349442 (PMC13178870; doi:10.1371/journal.pone.0349442)
Supplement: S1 Table — (DOCX) [file pone.0349442.s001.docx]

| **Variables** | **VIF** | **Variables** | **VIF** |
| --- | --- | --- | --- |
| HGS asymmtry(Method A) | 1.055 | HGS asymmtry(Method B) | 1.064 |
| Age | 2.177 | Age | 2.168 |
| Sex | 3.267 | Sex | 3.267 |
| Chlorpromazine equivalent dose | 1.222 | Chlorpromazine equivalent dose | 1.223 |
| Disease duration | 2.010 | Disease duration | 1.976 |
| BMI | 1.098 | BMI | 1.109 |
| MoCA-C scores | 2.167 | MoCA-C scores | 2.190 |
| PANSS scores | 1.689 | PANSS scores | 1.685 |
| Education | 1.491 | Education | 1.493 |
| Smoking history | 1.153 | Smoking history | 1.155 |
| Drinking history | 2.014 | Drinking history | 2.016 |

**S1 Table.** Variance Inflation Factor (VIF) for Multicollinearity Diagnostics of Independent Variables.
